# Accuracy of Emergency Medical Services Dispatcher and Crew Diagnosis of Stroke in Clinical Practice

**DOI:** 10.3389/fneur.2017.00466

**Published:** 2017-09-14

**Authors:** Judy Jia, Roger Band, Michael E. Abboud, William Pajerowski, Michelle Guo, Guy David, C. Crawford Mechem, Steven R. Messé, Brendan G. Carr, Michael T. Mullen

**Affiliations:** ^1^Department of Neurology, University of Pennsylvania, Philadelphia, PA, United States; ^2^Department of Emergency Medicine, Thomas Jefferson University, Philadelphia, PA, United States; ^3^Massachusetts General Hospital, Department of Emergency Medicine, Boston, MA, United States; ^4^Brigham and Women’s Hospital, Department of Emergency Medicine, Boston, MA, United States; ^5^Department of Healthcare Management, Wharton School, University of Pennsylvania, Philadelphia, PA, United States; ^6^Leonard Davis Institute of Health Economics, University of Pennsylvania, Philadelphia, PA, United States; ^7^Department of Emergency Medicine, University of Pennsylvania, Philadelphia, PA, United States; ^8^Philadelphia Fire Department, Philadelphia, PA, United States

**Keywords:** ischemic stroke, intracranial hemorrhage, transient ischemic attack, stroke systems, emergency medical services, prehospital, sensitivity

## Abstract

**Background:**

Accurate recognition of stroke symptoms by Emergency Medical Services (EMS) is necessary for timely care of acute stroke patients. We assessed the accuracy of stroke diagnosis by EMS in clinical practice in a major US city.

**Methods and results:**

Philadelphia Fire Department data were merged with data from a single comprehensive stroke center to identify patients diagnosed with stroke or TIA from 9/2009 to 10/2012. Sensitivity and positive predictive value (PPV) were calculated. Multivariable logistic regression identified variables associated with correct EMS diagnosis. There were 709 total cases, with 400 having a discharge diagnosis of stroke or TIA. EMS crew sensitivity was 57.5% and PPV was 69.1%. EMS crew identified 80.2% of strokes with National Institutes of Health Stroke Scale (NIHSS) ≥5 and symptom duration <6 h. In a multivariable model, correct EMS crew diagnosis was positively associated with NIHSS (NIHSS 5–9, OR 2.62, 95% CI 1.41–4.89; NIHSS ≥10, OR 4.56, 95% CI 2.29–9.09) and weakness (OR 2.28, 95% CI 1.35–3.85), and negatively associated with symptom duration >270 min (OR 0.41, 95% CI 0.25–0.68). EMS dispatchers identified 90 stroke cases that the EMS crew missed. EMS dispatcher or crew identified stroke with sensitivity of 80% and PPV of 50.9%, and EMS dispatcher or crew identified 90.5% of patients with NIHSS ≥5 and symptom duration <6 h.

**Conclusion:**

Prehospital diagnosis of stroke has limited sensitivity, resulting in a high proportion of missed stroke cases. Dispatchers identified many strokes that EMS crews did not. Incorporating EMS dispatcher impression into regional protocols may maximize the effectiveness of hospital destination selection and pre-notification.

## Introduction

To be maximally effective, stroke therapies, including tissue plasminogen activator (rt-PA) and endovascular thrombectomy (ET), must be delivered as quickly as possible (1). The American Heart Association recommended development of regionalized systems of care, preferentially transporting patients to the nearest stroke center, rather than the nearest hospital (2, 3). These recommendations are being adopted across the US (4). The impetus to bring patients with severe stroke directly to a Comprehensive Stroke Center is particularly pressing given randomized trials showing benefit of endovascular therapy (1).

Regionalized systems of care are dependent on early and accurate identification of stroke patients by Emergency Medical Services (EMS). Although validated prehospital stroke scales exist, the diagnostic sensitivity of EMS varies from 44 to 72% in clinical practice (5–9). We aimed to determine prehospital diagnostic accuracy of EMS dispatchers and crews for stroke overall, for acute stroke patients with National Institutes of Health Stroke Scale (NIHSS) ≥5, and which clinical features were associated with correct prehospital identification of stroke.

## Materials and Methods

We performed a retrospective observational study using data from September 2009 to October 2012 comparing prehospital diagnosis to discharge diagnosis of patients arriving *via* EMS to the Hospital of the University of Pennsylvania (HUP). The study protocol was approved by the Hospital of the University of Pennsylvania Institutional Review Board.

We matched patients identified as potential stroke in the Philadelphia Fire Department (PFD) database, a repository of clinical information for every 9–1–1 EMS encounter in Philadelphia, with confirmed stroke or TIA in the HUP Get with the Guidelines (GWTG) database, using patient identifiers, location, time of call, dispatcher impression, crew impression, and patient care report narratives. The PFD is the sole 9–1–1 EMS response agency for the City of Philadelphia. Patients who did not arrive *via* EMS or who could not be cross-matched were excluded. We recorded admission NIHSS, symptom duration on arrival, thrombolytic treatment, and discharge disposition. Final diagnosis was classified as TIA, infarct, hemorrhage, stroke mimic, or other. Stroke mimics included: seizure, hypoglycemia, intracranial tumor, spinal cord pathology, encephalopathy, migraine, recrudescence of previous stroke symptoms, and neuropathies.

Emergency Medical Services diagnosis and discharge diagnosis were compared to determine dispatcher and crew sensitivity and positive predictive values (PPV) for all stroke and TIA patients and separately for patients with NIHSS ≥5 presenting <6 h of symptom onset, to represent the cohort most likely to be eligible for rt-PA and ET. We calculated the number of stroke or TIA patients who were correctly identified by both EMS crew and dispatcher, and by each group alone. We created a logistic regression model including age, sex, stroke type (infarct versus hemorrhagic), NIHSS (<5, 5–9, 1 ≥ 0), clinical features (weakness, speech difficulty, altered mental status, ataxia, vision loss, and neglect determined by retrospective chart review), and symptom duration (<180, 180–270, and >270 min).

In a secondary analysis, we evaluated for differences between subjects with a vascular event who were correctly identified by dispatchers, but not EMS crews, and all other subjects with a vascular event. Pearson’s chi-squared test was used to examine for differences based on age, sex, stroke type (infarct versus hemorrhagic), NIHSS (<5, 5–9, 10≥), clinical features (weakness, speech difficulty, altered mental status, ataxia, vision loss, and neglect determined by retrospective chart review), and symptom duration (<180, 180–270, and >270 min). Variables were then included in a multivariable model.

## Results

**Figure 1:**
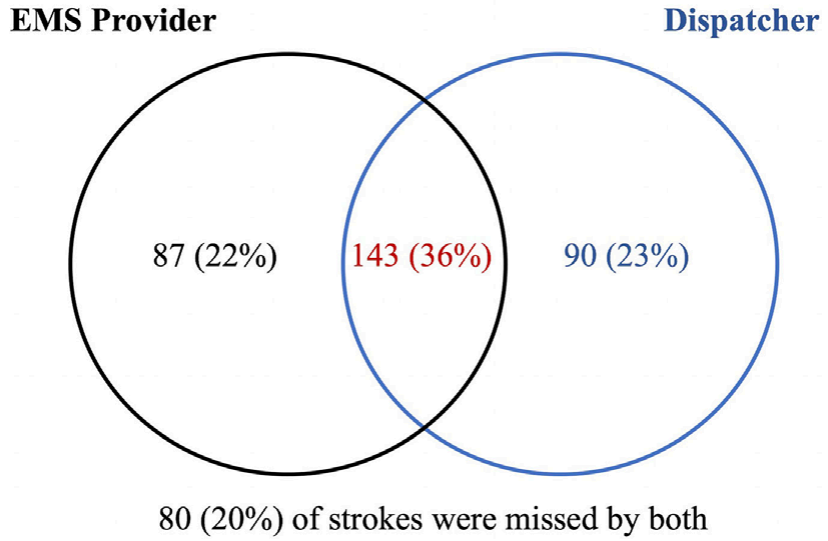
Overlap between emergency medical services (EMS) crew and dispatcher stroke identification. Of the 400 patients with a confirmed vascular event, 87 (22%) were correctly identified by the EMS crew alone, 90 (23%) by the dispatcher alone, and 143 (36%) by both.

There were 725 suspected stroke patients transported by the PFD to HUP from September 2009 to October 2012. Of these, 592 were confirmed to have had a stroke or TIA based on the GWTG registry. The final cross-referenced database contained 709 cases. There were 16 cases in the PFD database excluded due to missing patient identifiers. Of the 709 subjects in the final database, 269 (37.9%) were ischemic, 73 (10.3%) hemorrhagic, 58 (8.2%) TIAs, 111 (15.7%) stroke mimics, and 198 (27.9%) other diagnoses. Four hundred (56.4%) had a cerebrovascular event (TIA, infarct, or hemorrhage). Of the cases that the EMS crews identified as stroke, 72% were stroke or stroke mimic. EMS crews correctly identified 230 of these 400 patients as suspected stroke, yielding a sensitivity of 57.5% and PPV of 69.1%. Using both dispatcher and crew impression, 320 were correctly identified, which increased sensitivity to 80% and reduced PPV to 50.9%. Of the 400 cases with a confirmed vascular event, 87 (22%) were identified by the EMS crew alone, 90 (23%) by dispatcher alone, and 143 (36%) were identified by both (Figure 1). Of the 126 acute stroke patients with symptom onset < 6 h and NIHSS ≥ 5, EMS crews identified 101 (80.2%). Dispatchers correctly identified an additional 13 (10.3%) of these cases (Figure 1).

In univariate analysis, hemorrhagic stroke, NIHSS, and symptom duration were associated with a correct diagnosis by EMS (Table 1). NIHSS, shorter symptom duration, and weakness were independently associated with a correct diagnosis in the multivariable model (Table 2).

**Table 1:** Univariate analysis of factors associated with a correct diagnosis of stroke by emergency medical services (EMS) crews.

	EMS crew Dx-stroke	EMS crew Dx-other	*P*-value
Number of subjects, *n*	230	170	
Age, median (IQR)	64 (54–79)	61 (51–73)	0.064
<50	14.4	19.4	0.124
50–74	54.4	57.7	
75+	31.3	22.9	
Male, %	49.4	43.0	0.206
Stroke type, %			0.005
TIA	10.4	20.0	
Ischemic	67.4	67.1	
Hemorrhagic	22.2	12.9	
National Institutes of Health Stroke Scale median (IQR)	8 (3–16)	3 (1–6)	<0.001
<5	30.9	65.7	
5–9	26.1	18.3	
10+	43.0	16.0	
Symptom onset to ED arrival, median (range), min	141.5 (58–661)	3 (78–1066)	<0.001
<180	55.3	39.6	0.002
180–270	8.3	6.5	
>270	36.4	53.8	
Clinical features			
Weakness	77.4	52.9	<0.001
Speech difficulty	41.3	30.6	0.028
Altered mental status	28.3	21.2	0.107
Ataxia	1.7	10.0	<0.001
Vision loss	4.4	6.5	0.347
Neglect	2.6	2.4	1.000

**Table 2:** Multivariable analysis of factors associated with a correct diagnosis of stroke by emergency medical services crews.

	OR	95% CI	*P*-value
Age			0.082
<50	Ref		
50–74	1.28	(0.67, 2.42)	0.456
75+	2.15	(1.03, 4.48)	0.041
Sex	1.37	(0.86, 2.17)	0.182
Stroke type			0.385
TIA	Ref		
Ischemic	1.16	(0.58, 2.33)	0.683
Hemorrhagic	1.8	(0.72, 4.54)	0.002
National Institutes of Health Stroke Scale			<0.001
<5	Ref		
5–9	2.62	(1.41, 4.89)	0.002
10+	4.56	(2.29, 9.09)	<0.001
Symptom onset to ED arrival, min			0.002
<180	Ref		
180–270	0.97	(0.38, 2.51)	0.953
>270	0.41	(0.25, 0.68)	0.001
Clinical feature			
Weakness	2.28	(1.35, 3.85)	0.002
Speech difficulty	1.48	(0.89, 2.46)	0.133
Altered mental status	0.75	(0.39, 1.44)	0.385
Ataxia	0.34	(0.10, 1.17)	0.087
Vision loss	0.56	(0.20, 1.60)	0.280
Neglect	0.47	(0.11, 1.92)	0.290

Comparing subjects with a vascular event who were identified by dispatchers alone to all other subjects in a univariate analysis, dispatchers alone were more likely to identify patients with TIA, low NIHSS, and vision loss at presentation; dispatchers were less likely to identify patients with altered mental status at presentation (Table 3). In a multivariable model, age, NIHSS, and vision loss were independently associated with identification by dispatchers but not EMS crews (Table 4).

**Table 3:** Univariate analysis of factors associated with correct identification by dispatchers only.

	Dispatcher only	All others	*P*-value
Number of subjects, *n*	90	310	
Age			0.102
<50	18.9%	15.9%	
50–74	62.2%	53.9%	
75+	18.9%	30.3%	
Male, %	42.2%	46.8%	0.445
Stroke type			0.001
TIA	24.4%	11.6%	
Ischemic	66.7%	67.4%	
Hemorrhagic	8.9%	21.0%	
National Institutes of Health Stroke Scale			<0.001
<5	66.3%	39.7%	
5–9	22.5%	22.9%	
10+	11.2%	37.4%	
Symptom onset to ED arrival			0.151
<180 min	40.5%	51.0%	
180–270 min	6.7%	7.8%	
>270 min	52.8%	41.2%	
Clinical feature			
Weakness	61.1%	68.7%	0.177
Speech difficulty	35.6%	37.1%	0.789
Altered mental status	12.2%	29.0%	0.001
Ataxia	7.8%	4.5%	0.222
Vision loss	10.0%	3.9%	0.022
Neglect	3.3%	2.3%	0.565

**Table 4:** Multivariable analysis of factors associated with correct identification by dispatchers only.

	OR	95% CI	*P*-value
Age			0.034
<50	Ref		
50–74	0.97	(0.49, 1.92)	0.933
75+	0.42	(0.18, 0.96)	0.04
Sex	1.22	(0.73, 2.05)	0.449
Stroke type			0.148
TIA	Ref		
Ischemic	0.6	(0.29, 1.23)	0.16
Hemorrhagic	0.35	(0.12, 1.02)	0.055
National Institutes of Health Stroke Scale			0.008
<5	Ref		
5–9	0.6	(0.30, 1.19)	0.144
10+	0.25	(0.10, 0.60)	0.002
Symptom onset to ED arrival			0.097
<180 min	Ref		
180–270 min	0.92	(0.31, 2.78)	0.887
>270 min	1.82	(1.03, 3.25)	0.041
Clinical feature			
Weakness	0.96	(0.53, 1.72)	0.881
Speech difficulty	1.1	(0.62, 1.96)	0.729
Altered mental status	0.86	(0.38, 1.91)	0.702
Ataxia	1.1	(0.39, 3.15)	0.852
Vision loss	3.74	(1.32, 10.62)	0.013
Neglect	3.75	(0.77, 18.2)	0.101

## Discussion

Intravenous rt-PA and ET have been shown to dramatically improve outcomes in eligible subjects. ET is not widely available, and transfer times between hospitals are often long (10, 11), so it is critical to identify stroke patients in the field and triage them to appropriate hospitals based on both diagnosis and suspected presence of a large vessel occlusion. Although scales exist to identify large vessel occlusion (12), EMS must first recognize that the patient is having a stroke.

The sensitivity of EMS crew identification of stroke was low at 57.5%. Philadelphia adheres to the Pennsylvania Statewide Advanced Life Support Protocols and Basic Life Support Protocols, which include the Cincinnati prehospital stroke scale (CPSS) for evaluation of suspected stroke patients (http://pehsc.org/wp-content/uploads/2015/06/Statewide_ALS_Protocols-2015-FINAL-06-01-15.pdf, http://pehsc.org/wp-content/uploads/2014/05/Statewide_BLS_Protocols_Final_020915.pdf). Although our results are lower than the CPSS sensitivity reported in some studies (13, 14), our results are similar to other studies in large metropolitan areas (5–9). This highlights the variability in EMS performance across geographic areas. Interestingly, we found that dispatchers identified 90 stroke patients that EMS crews did not. However, the collective dispatcher and EMS crew sensitivity exceeded that of previously reported EMS sensitivity. This builds on prior data, which showed dispatchers had greater diagnostic sensitivity than EMS crews (15). Civilians calling to report a suspected stroke are often correct in their initial impression (16), and it is possible that they raise the dispatcher’s suspicion for stroke. EMS dispatchers in Philadelphia perform a verbal FAST screen (face, arm, speech, time) in accordance with their proprietary emergency medical dispatching software. Differences in compliance with prehospital stroke scales among dispatchers and crews or changes in symptoms over time could also account for these findings.

Our finding that patients who were identified by dispatchers alone were more likely to have TIA, low NIHSS, and vision problems suggest that subtle and/or improving symptoms may be a factor in the discrepancy between EMS crew and dispatcher diagnosis. However, a combined approach to identification of stroke, which identified ischemic stroke that was identified by either the dispatcher or the EMS crew, was the most sensitive for identification of strokes with symptom duration <6 h and NIHSS ≥5. Given this, it seems that incorporating dispatcher impression into EMS prenotification protocols may be a viable option to improve prehospital recognition of stroke and maximize the use of rt-PA and ET. On the other hand, our data also illustrate the trade-offs of such an approach. Using EMS crew or dispatcher impression increased sensitivity, but PPV dropped from 69.1 to 50.9%. This suggests that a protocol incorporating both crew and dispatcher impression would increase the proportion of patients without stroke who were incorrectly triaged to a stroke center, which could be detrimental, particularly if local stroke centers are already operating near maximum capacity.

The retrospective nature of our single-center chart review has limitations. First, although Pennsylvania statewide EMS protocols include the CPSS for evaluation of suspected stroke patients, the database does not record whether this scale was used. As a result, we could not analyze how the use of a prehospital stroke scale affected EMS diagnostic accuracy. Second, HUP was a certified stroke center during the study period, so, EMS crews may have triaged more definitive, severe strokes to our center. If true, we may have overestimated EMS diagnostic accuracy. A third limitation was that we could not distinguish whether an ALS or BLS ambulance was dispatched on a particular call. While the paramedics staffing ALS ambulances function full-time in that role, during the study period, BLS ambulances were staffed with firefighter-EMTs who would work in an ambulance for several shifts, then rotate back to fire apparatus. As a consequence, their diagnostic accuracy may not have been comparable to that of paramedics. Finally, we also assumed that the database input fields truly reflect dispatcher and crew clinical impression. Given the time pressures facing EMS, recorded diagnostic impressions may not be an accurate assessment of their clinical suspicion and thus underestimate their diagnostic sensitivity.

Variability in prehospital identification of stroke across the US may limit the effectiveness of regionalized systems of care. Focused training initiatives are needed to improve EMS recognition of stroke and to ensure the use of prehospital stroke scales. Because dispatchers in this study identified stroke patients that crews did not, incorporating dispatcher diagnosis into prehospital interventions, EMS prenotification, or prehospital telemedicine, may increase the impact of these interventions.

## Ethics Statement

The study protocol was approved by the Hospital of the University of Pennsylvania Institutional Review Board.

## Author Contributions

JJ: protocol development, data abstraction, data analysis, data interpretation, manuscript drafting/revising. RB: protocol development, data interpretation, manuscript drafting/revising. MA: data abstraction, data analysis, manuscript drafting/revising. WP: data analysis, manuscript drafting/revising. MG: data abstraction, manuscript drafting/revising. GD: protocol development, data interpretation, manuscript drafting/revising. CM and SM: data interpretation, manuscript drafting/revising. BC: protocol development, data interpretation, manuscript drafting/revising. MM: protocol development, data abstraction, data analysis, data interpretation, manuscript drafting/revising.

## Conflict of Interest Statement

The authors declare that the research was conducted in the absence of any commercial or financial relationships that could be construed as a potential conflict of interest. The reviewer, PC, and handling editor declared their shared affiliation.
